# The Built Environment—A Missing “Cause of the Causes” of Non-Communicable Diseases

**DOI:** 10.3390/ijerph13100956

**Published:** 2016-09-27

**Authors:** Kelvin L. Walls, Mikael Boulic, John W. D. Boddy

**Affiliations:** 1 Building Code Consultants Ltd., P.O. Box 99613, Newmarket, Auckland 1149, New Zealand; 2 School of Engineering and Advanced Technology, Massey University, Auckland 0745, New Zealand; m.boulic@massey.ac.nz; 3 Urban Planning and Environmental Services, MWH Stantec, Level 3, 111 Carlton Gore Road, Auckland 0745, New Zealand; Doug.Boddy@mwhglobal.com

**Keywords:** United Nations 25 × 25 Strategy, non-communicable diseases (NCDs), built environment, hazardous building materials, involuntary causes, global NCD burden, missing NCDs, missing causes, environmental factors, sick building syndrome

## Abstract

The United Nations “25 × 25 Strategy” of decreasing non-communicable diseases (NCDs), including cardiovascular diseases, diabetes, cancer and chronic respiratory diseases, by 25% by 2025 does not appear to take into account all causes of NCDs. Its focus is on a few diseases, which are often linked with life-style factors with “voluntary” “modifiable behavioral risk factors” causes tending towards an over-simplification of the issues. We propose to add some aspects of our built environment related to hazardous building materials, and detailed form of the construction of infrastructure and buildings, which we think are some of the missing causes of NCDs. Some of these could be termed “involuntary causes”, as they relate to factors that are beyond the control of the general public.

## 1. Introduction

The World Health Organization (WHO) estimated that, in 2012, 68% of the global deaths were due to non-communicable diseases (NCDs) [1]. Pearce et al. [2] commented on the approach to addressing the global NCD burden—the “25 × 25 Strategy”—promulgated by the UN high-level meeting on NCD, of 2011, and the 2012 World Health Assembly [3]. This strategy emphasizes four diseases (cardiovascular disease, diabetes, cancer, and chronic respiratory disease), which account for 87% of all NCDs deaths, and four key risk factors (tobacco use, unhealthy diet, lack of physical activity, and overuse of alcohol). However, a different picture of disease and risk factors emerges if the burden of morbidity is also considered.

The “big four” NCDs account for only 54% of disability-adjusted life years (DALYs) which incorporate information on mortality and morbidity [2]. Other important NCDs include neurological disease (including Alzheimer’s disease), mental health disorders, musculoskeletal disorders, and hearing and vision loss. Pearce et al. (2014) [2] also suggested that the 25 × 25 Strategy overlooks diseases, such as asthma, which falls under the umbrella of respiratory diseases, but, whilst rarely fatal, nevertheless, contributes a significant proportion of DALYs.

The “missing” NCDs are not generally caused by lifestyle factors, such as tobacco use, harmful use of alcohol, unhealthy diet, and lack of physical activity, as targeted by the 25 × 25 Strategy [2]. Instead, these “missing causes” likely include adverse health exposures caused by our built environment, which could be occupational exposures and residential exposures. Infections and occupational and environmental exposures may be other key “missing causes”. They further describe the “25 × 25 Strategy” as avoiding complexity, using narrowly defined targets and ignoring “what is occurring outside of its simplified framework” [4].

Key structural determinants of these missing causes, sometimes termed the “causes of the causes”, include urban design (“Urban design”, as discussed by Pearce et al., 2014 [2] appears to consider the larger urban environment and aspects like traffic pollution, in contrast to our approach of the “built environment” considering buildings and infrastructure that enclose and house people), poverty and development, air pollution (associated with greater than six million deaths annually), lifestyle and climate change [2] and greater than seven million deaths per year worldwide (from ambient and household air pollution) [5]. Pearce et al. (2005) [4] suggest the 25 × 25 Strategy is an over-simplification of a much more complex situation.

We further develop this view by suggesting that some more micro aspects of our built environment may themselves be important factors contributing to NCDs. These may include the influence of the outdoor contaminants on indoor environments like particulate matter from combustion processes (vehicle, industry) [6], hazardous building materials, and include potential adverse health concerns like exposure to particulate matter, by-products of burning fossil fuels, fiberglass, asbestos, high/low humidity, radon, heavy metals, chemicals like formaldehyde, hormones and endocrine-disrupting chemicals (EDCs) and copper in water supplies. These may arguably be termed “involuntary” origins of toxins that lead to many NCDs. The built environment is a missing cause of the targeted NCDs in the 25 × 25 Strategy, as well as being a likely cause of the missing NCDs. We describe an important, but overlooked, missing “cause of the causes” related to urban design—The more micro aspects of the urban environment, which we call the “built environment”.

## 2. The Built Environment as a Missing “Cause of the Causes” 

It is widely accepted that the general environment in which we live contributes to chronic disease—25% to 35% of the global burden of disease [7]. Some researchers have suggested strong links of modern environmental factors with inflammation and chronic disease [8].

While chronic disease risk is reported to be caused by a combination of genetic and environmental factors, up to 90% of disease risk could be attributed to environmental factors (The estimate of 25%–35% by Bonita et al., 2006 [7] is noted in contrast to that of up to about 90% by Rappaport and Smith, 2010) [9]. This adds difficulties to analyze as the traditional “silo” approach of epidemiologists specializing in specific categories, such as air and water pollution, occupational hazards, diet and obesity, stress and behavior, for example, leads to confusion regarding the definition and more general understanding of “environmental exposures” [9]. A collective investigation of these exposure categories would also help elucidate possible additive or synergistic effects of combinations of pollutants, such as between the inhalation of asbestos fibers and cigarette smoking [10]. However, the impact from multiple potential sources is difficult to establish and complex to untangle.

Anthropogens are man-made “environments/products”. The by-products and/or the lifestyles encouraged by those anthropogens could have metabolic effects that may be detrimental to human health [11]. Post-industrial environments have led to anthropogens being formed and sometimes with resultant metaflammation (which is related to metabolically triggered inflammation but without infection). Some of these environments generate particulate matter (PM) and general air pollution (which contribute to cancer mortality [12,13], traffic-related air pollution, water pollution and EDCs—called *obesogens* because of their possible link to obesity [11].

Anthropogen building materials/built environments do not always have adverse health effects. However, some known unhealthy anthropogens (contaminants) include asbestos in building materials [11], low humidity “sick building” syndrome [8] and also high humidity built environments, which are more conducive to the growth of bio-contaminants like fungi and bacteria. Fiberglass used for insulating buildings and formaldehyde used in many manufactured building boards and furnishings are likely to be potentially harmful anthropogens.

Air pollution, such as welding fumes and fumes from unflued gas appliances, are able to contribute indirectly to chronic disease (in non-smokers) via several functional pathways including oxidative stress and pro-inflammatory responses [14]. There are dangers of smoke exposure from the indoor use of unflued gas cooking and heating, which exacerbates lung and respiratory conditions, especially in low income populations more dependent on this type of fuel use for cooking and heating [15,16]. There are likely about 3.5 million deaths annually from household air pollution from unvented solid fuel burners [17].

A great deal of evidence has been produced linking general air pollutants and PM with various chronic diseases [18,19] and so we shall focus on some of the micro situations of our built environment which are likely etiological factors of NCDs and are further “missing” causes of NCDs.

## 3. Unflued Combustion Effluents for Cooking and Heating

The combustion of natural gas leads to indoor air pollution from gas ranges, ovens, pilot lights, gas and kerosene space heaters, which contribute to nitrogen oxide (NO), nitrogen dioxide (NO_2_), carbon monoxide (CO) and, for kerosene space heaters, sulfur dioxide (SO_2_) pollution [20,21,22], as well as over 300 volatile organic compounds (VOCs) although they are not all present at one time, with formaldehyde (CH_2_O) as the most prevalent [20].

Space heating appliances using natural gas (mostly methane), LPG (60% propane, 40% butane) and kerosene release by-products during combustion (mostly carbon dioxide (CO_2_) and water), are shown in the following complete combustion reaction equation:

CH_4_ + 2O_2_ → CO_2_ + 2H_2_O + other by products like CO, HCHO, SO_2_, NO_2_ + heat (1)

Additional impurities and additives in methane, propane, butane and kerosene may include radon and other radioactive materials. The components of the gas itself and the by-products of incomplete combustion, such as NO_2_, CO and others, have health implications individually and synergistically, along with those of other indoor pollutants [23].

Bettany et al. (1993) [24] reported NO_2_ levels between 69 ppb and 280 ppb in households operating unflued gas heaters (UGH), which is close to three times the WHO recommended value of 200 µg/m³ or 106 pbb [25]. Similar results of 190 ppb to 930 ppb were reported by Ferrari et al. (2004) [26] (8.5 times the WHO recommended value). In addition, chamber testing following UGH manufacturers’ instructions found values from four to eight times above the recommended value [27,28]. Gillespie-Bennett et al. (2008) [29] found a NO_2_ level three times higher in households operating an UGH compared with the households operating heat pumps or plug-in electric heaters. Kingham and Petrovic (2005) [30] found a NO_2_ level 3.8 times higher in eight households operating UGH compared with eight households operating electric heaters. Farrar et al. (2005) [31] found a NO_2_ level two times higher in households operating UGH than in those using non-gas heaters.

Ferrari et al. (2004) [28], Francisco et al. (2010) [32] and Upton et al. (2004) [28] also found high CO levels in households using UGH.

The adverse health effects of indoor air pollutants depend on the exposure period, as well as the health status and the age of the person exposed to the pollutant. Elderly, infants, young children, pregnant women and people with a disease (asthma, bronchitis, chronic obstructive pulmonary disease) are more vulnerable than other people, for the same level of exposure [33]. There is positive evidence linking gas stoves for cooking and other unvented gas appliances with respiratory symptoms in women [34].

The International Study of Asthma and Allergies in Childhood (ISAAC) study by Wong et al. (2013) [35] concluded that while the use of open fires for cooking is associated with asthma-related problems in children there is no evidence linking respiratory symptoms in children with the use of gas cooking in homes. Those who carry out most of the cooking (women) are more prone to adverse health conditions [36]. However, strong evidence of relationships between unflued combustion of cooking appliances and higher indoor levels of pollutants has been reported [37]. Furthermore, there were differences in secondary outcomes, such as a reduction in difficulty breathing, decreased chest tightness and reduction of asthma attacks when unflued gas heaters were replaced with flued gas heaters or electric heaters [38]. Less disturbed sleep from wheezing and less dry cough at night were reported when UGHs were replaced with a heat pump, flued gas heater or wood pellet burner [39].

Overall, the literature shows strong evidence of relationships between unflued combustion and reported exposure to high indoor levels of pollutants (CO_2_, CO, CH_2_O and NO_2_) in homes. Built environment activities like unflued cooking or unflued heating could, therefore, be considered as causes of NCDs.

## 4. Environmental Factors Related to Sick Building Syndrome

“The term “sick building syndrome” (SBS) is used to describe situations in which building occupants experience acute health and comfort effects that appear to be linked to time spent in a building, but no specific illness or cause can be identified.” [40].

We spend over 70% of our time inside buildings with little awareness as to how indoor environments may adversely affect public health, such as exposure to known factors of dust mites, endotoxins, mold and passive smoke inhalation [41].

While sick building syndrome (SBS) is often focused on biological contaminants, it is also related to other factors like chemical contaminants, inadequate ventilation, poor lighting and poor acoustics [42].

Viable mold spores are ubiquitous, and thus always present in homes. Temperature, moisture and nutrients are critical factors for spore germination, hyphae development and sporulation [43]. Building materials and furniture are potential sources of nutrients, substrates for fungi colonization and other principal determinants of the fungal activity [44,45]. The use of fiberglass insulation lining air handling systems can be a cause of contamination if the fiberglass becomes wet or dirty, leading to conditions that promote the growth of bacteria, mold and fungi, unless suitable regular maintenance is carried out [46]. Fungal colonization could lead to material degradation and human allergen production [47]. Furthermore, spores, fragments of mycelium, mycotoxins and microbial volatile organic compounds could be harmful to people, particularly immune deficient people or people with asthma [48,49]. Occupational asthma is a leading cause of work loss in the U.S. [50] contributing to DALYs of NCDs. Indoor exposure to CO_2_ on workers can impair performance and decision-making [51] but this raises a number of questions for managing indoor air quality in offices, schools, airplanes, cars and homes.

A major consideration arising from the variable nature and use of buildings is indoor relative humidity (RH), which is linked with the presence of fungi within the building [52,53]). The use of UGH can increase RH to high and unhealthy levels, but the mechanisms linking the specific causal dampness and the related agents are still not clarified [54]. Mold and other microbiological organisms are probably the link between dampness in buildings and adverse health effects [55].

Low humidity “sick building” syndrome is linked with metaflammation [8]. Some air conditioning systems cause this environmental condition.

The fungi, *Aspergillus*, play an important role in some organic dust diseases [56]. The genus *Aspergillus* is found worldwide, and is one of the most common groups of all fungi [57,58,59] *Aspergillus fumigatus* is a dominant agent causing allergic broncho-pulmonary aspergillosis [57,60,61]. Aspergillosis is the second most common fungal infection in the U.S. requiring hospitalization. There is evidence supporting links with aspergilli and other fungi in relation to SBS [57].

A common link with buildings and potential NCDs is that *Aspergillus* species are a common microorganism on ceiling tiles, insulation, painted surfaces and wallpaper. However, the worst effects of fungi infection occur only in a few individuals who have immuno-suppressed host defenses [62].

Dampness in buildings increases the capacity for fungi like *Aspergillus* to grow and fungi are known to cause allergic reactions [63]. Fungi in heating and ventilating air conditioning systems may play a significant role in some “sick” buildings [64]. Sand, soot and fiber trash inside ducts favor fungal growth, which could contaminate indoor environments [65].

## 5. Fiberglass

The smaller the particle, the deeper it will migrate into the respiratory tract during breathing. While glass wool insulation fibers are between 5 µm and 10 µm (PM_10_) in diameter, a small proportion of fibers are less than 2.5 µm (PM_2.5_), allowing them to be breathed deep into the lungs. Along with binding resins and oils they can cause irritation [66]. Fibers of some special purpose fiberglass can be 1 µm in diameter [46], which could make them an extra health hazard due to their small size, in the absence of mitigation. The risks apply mostly to construction workers or home renovators during installation of insulation. There is also a likelihood of risks to occupants of fiberglass particles passing through ceilings that are not formed of continuous sheets and have gaps between boards.

Although in 1990 it was reported that there was no epidemiological evidence of human disease from the inhalation of conventional glass fibers [67], the International Agency for Research on Cancer (IARC) until November 2001 considered fiberglass as a possible carcinogen but this classification changed so that it is no longer considered to be the case. This is because of studies showing no evidence of increased risk due to occupational exposure and that there is an increased use of biosoluble fiberglass [66].

While fiberglass is considered to be a harmful anthropogen, we note that there is a lack of consensus as to whether exposure to its fibers is carcinogenic, but there may be more evidence to link inhalation of fiberglass particles with NCD morbidity DALYs. Furthermore, according to WHO (2002) [68], there is no safe exposure threshold for the inhalation of particles smaller than 2.5 µm (PM_2.5_), so even if it is not carcinogenic, exposure to high PM_2.5_ numbers (particle counts) and concentrations have the potential to lead to adverse health effects [69,70].

While some air conditioning systems can exacerbate conditions of SBS, they are a tool in reducing indoor particulate matter. The British Standard for air filters [71] provides for air filters used in HVAC systems to reduce indoor concentrations of PM_2.5_ [72] as do standards of other jurisdictions, such as Australia and North America.

## 6. Asbestos

Asbestos is a strong and flexible fibrous mineral with good fire and chemical resistance that was widely used in the 20th century as building insulation, as part of construction materials, in concrete and as an additive to paint and sealants [73]. Due to safety concerns its use in building materials ceased in about 1981 [74]. This presumably relates to the situation in the U.S. and some other countries as it is still used in a large number of countries where there is often little or no protection for workers and communities [75]. It has long been known that inhalation of asbestos fibers can result in a variety of respiratory diseases [76]. Asbestosis usually occurs after prolonged and intensive exposure to asbestos but pleural mesothelioma may occur following only brief exposures [77].

The risks of NCDs associated with exposure to asbestos have been well researched and accepted by the international community generally; albeit that asbestos is still used in many countries.

A point to note is that in a home that contains asbestos, the material may or may not pose a health hazard, depending on its condition. If the surface is stable, undamaged, and well-sealed against the release of its fibers, it is usually considered safe. However, when the surface is not sealed, or it can be crushed by hand pressure, or it is already loose (such as with vermiculite insulation), fibers can be released and may pose a health risk, including lung cancer. In these cases, asbestos should only be removed by a professional (thus reducing the exposure risk to the general public/home owners).

Asbestos is attributed to around 194,000 deaths and 3,402,000 DALYs in 2013, making it responsible for nearly two-thirds of the burden of all occupational carcinogens [78]. Despite the well-known danger of asbestos its use is not declining but moving and increasing in low-income countries. In India, for example, its use has doubled to about 300,000 tons per year in the last decade and the industry employs about 100,000 workers [4].

## 7. Formaldehyde

Formaldehyde (CH_2_O) is the most common and best-known indoor air pollutant [79] attributed to unvented fuel-burning (kerosene, LPG) appliances [80]. In the urban environment, the main source of CH_2_O is related to fossil-fuel combustion from motor vehicles and domestic solid-fuel combustion [81]. An unvented gas heater on high setting with a 0.5 ACH ventilation rate caused a CH_2_O level above WHO recommendations [28] being 0.1 mg/m³ or 0.08 ppm [25]. The same study showed that even when following the UGH manufacturer’s recommendations [82], the CH_2_O level exceeded the maximum recommended level by 2.7 times. More comprehensive testing found that the CH_2_O level during UGH operation was 2.5 times higher [26].

CH_2_O used in many home furnishings, household cleaners, paints, textiles, medicinal and personal care products [83] manufactured building boards and insulation is a potentially harmful anthropogen and carcinogen [84] the dangers of which came to the fore in 1980 [79]. In a study of New Zealand houses, formaldehyde levels exceeded WHO’s recommendation of 0.08 ppm as a 30 min average in 84% of the monitored living rooms and bedrooms with one home with a CH_2_O level of 1.8 ppm which coincided with unflued gas combustion [85]. Due to its common use numerous workers in industrial situations are exposed to particularly high concentrations of formaldehyde [86]. While it is suspected to be a causal agent of certain types of cancer, there is only limited evidence of the carcinogenic effects on humans [86,87]. It is also a natural occurring substance of less than 0.03 ppm [83]. A wide range of manufactured wood products, paints, wallpaper, carpet and insulation materials contain formaldehyde, which may lead to excessive indoor levels of the contaminant for the first few weeks of habitation. Very hazardous levels of up to 3.68 ppm were found in homes with higher levels in newly manufactured or mobile homes [87]. The most significant source of formaldehyde in homes is from pressed wood products using urea-formaldehyde (UF) resins [80,87].

Wood and construction related industries use 60% of worldwide production of formaldehyde. Hazardous levels are used in fumigants against rodents and other pests inside buildings [88]. Despite the known hazards, it is still often used as a binder in fiberglass insulation [89].

Some immediate symptoms of exposure to formaldehyde may include eye, throat and skin irritation, coughing, wheezing, asthma, allergic reactions and neurological health effects with longer term chronic conditions including cancer [83,84,90]. The first acute effects of formaldehyde are usually to the eyes, nose and throat, which if prolonged can lead to alteration of pulmonary function, asthma and bronchitis [86].

## 8. Radon

Air pollution caused by radon is ubiquitous in our environments; particularly our indoor environments [91,92,93,94] and radon can accumulate inside buildings, especially if they are poorly ventilated.

Radon is one of the most extensively investigated human carcinogens [95]. Worldwide, it is the second cause of lung cancer in the general population after smoking [96,97].

In the drive to make buildings more energy efficient, which includes making them better sealed against draughts and unwanted air infiltration [98], energy efficient buildings need to be provided with adequate ventilation to avoid elevated concentrations of indoor pollutants such as radon [99], but also CO_2_ and other contaminants.

## 9. Heavy Metals, Chemicals, Hormones and Endocrine-Disrupting Chemicals

Apart from the difficulties that many microbiological contaminants pose in relation to assessing the safety of potable water, there are also an increasing number of heavy metals [100] and EDCs contaminating human effluent [101]. Epidemiological studies show that environmental exposures to low doses of EDCs are associated with human disease and disability [100,102,103].

Vandenberg et al., 2012 [103] list 28 EDCs with reported low-dose effects in animals or humans. A U.S. Federal study found 112 toxic materials from everyday life are reaching the Columbia River after having passed through wastewater treatment plants, including flame retardants, pharmaceuticals, pesticides, personal care products, mercury, and cleaning products.

All human sewage (wastewater) contains many of these “low-dose” compounds of concern. There is no known sustainable, septic or sewage treatment system, existing or in concept, that can reliably neutralize (or even identify) these compounds. There is a need for changes in chemical testing and safety determination in order to protect human health [103]. This need becomes paramount with an increasing reuse of wastewater as potable water [104].

Carcinogenic, hormonal, heavy metal and radiological effects must be considered for treatment of recycled water for drinking purposes [105,106] but chronic effects to low levels of chemicals are not well understood, requiring further research [106].

## 10. Copper in Water Supplies

Alzheimer’s disease and other NCDs are linked with excess copper consumption (from water supplies), Dissolved copper in water from copper supply pipes and fittings in buildings is a contaminant with potential adverse health effects [107,108,109].

Water acidity is problematic given that copper water supply pipes are commonly used. This could be an issue for those living off town water supplies that do not routinely control pH, and also for private, on-site rainwater tanks, where pH level controls are not normally applied. The more acidic water is likely to lead to greater corrosion of copper water pipes in buildings [110].

Copper is both an essential micronutrient and a drinking water contaminant—too little leads to a physiological deficiency, whilst too much causes toxicity [107,111,112] including liver disease [113,114,115].

Excess copper in the body reportedly accumulates in the liver, brain and eyes. Some studies have suggested that accumulation of copper in the eyes and brain can lead to age-related macular degeneration (a common form of blindness in older people) [116,117,118] and Alzheimer’s disease [115,119,120]. There is not full agreement over this view, however, as some researchers believe that a direct link cannot be made between copper exposure and Alzheimer’s Disease [121].

## 11. Conclusions

The human environment, and indeed the built environment, are far different from those of our early ancestors and have been developed incrementally (and exponentially in recent years) using new technologies. There is no doubt that it has led to a more comfortable and healthy life and has contributed to our increased longevity. However, with all life changes using new technologies there can be unintended consequences. Checks and balances need to form an integral part of the technological evolution of mankind.

In this paper, we have explored some current technologies that appear to have a distal link with metaflammation and chronic disease (NCD), resulting predominately (but not exclusively) from PM inhalation. The anthropogenic sources of such pollution are additive to naturally occurring sources such as volcanoes, fires, dust storms and aerosolized sea salt.

When new innovations are put to prolific use, such as occurred with asbestos over a long period of time, we must not only narrowly consider the beneficial attributes of the product, but we must also be circumspect enough to stand back and critically consider any adverse consequences that may be faced with the use of the product.

The UN General Assembly is working on its 25 × 25 Strategy, which focuses of the four most dominant NCDs (“voluntary” causes) with which it is suggested that some life style changes can influence the required reduction in NCDs. We suggest therefore, that the four most prominent NCDs are “voluntary” (modifiable behavioral risk factors) in relation to their etiology.

However, we suggest that some further “missing” causes derive from unintended NCDs from toxins within our built environment (including, but not limited to, gas space heating, sick building syndrome, fiberglass, asbestos, formaldehyde, radon, heavy metals, chemicals, hormones and EDCs and copper in water supplies) (“involuntary” causes) and that these are worthy of further investigation and research.
